# 747. Employer requirements of COVID-19 vaccination reported by healthcare personnel, April 2022

**DOI:** 10.1093/ofid/ofad500.808

**Published:** 2023-11-27

**Authors:** Marie de Perio, Anup Srivastav, Anthony Laney, Carla Black, Hilda Razzaghi

**Affiliations:** Centers for Disease Control and Prevention, Cincinnati, Ohio; Leidos Inc, Atlanta, Georgia; National Institute for Occupational Safety and Health, Morgantown, West Virginia; CDC, Atlanta, Georgia; CDC, Atlanta, Georgia

## Abstract

**Background:**

Healthcare personnel (HCP) are at risk for acquiring and transmitting coronavirus disease 2019 (COVID-19) at work. Vaccination against COVID-19 is a primary strategy to prevent transmission in healthcare settings. Our objectives were to describe HCP reporting employer COVID-19 vaccine requirements, identify differences across occupations and settings, and describe policies.

**Methods:**

In a national opt-in internet panel survey of HCP in April 2022 to assess influenza and COVID-19 vaccination coverage, respondents were also asked about employer vaccination requirements. We weighted responses to the U.S. HCP population by age, sex, race, ethnicity, work setting, and U.S. census region. We assessed differences using two-tailed t tests with significance set at p< 0.05. We used multivariable logistic regression to identify factors associated with reporting employer requirements.

**Results:**

Of 3,618 responding HCP, 60.0% reported employer requirements for the COVID-19 primary series. Of those, 95.5% reported completing the primary series vs. 81.9% without requirements (p< 0.001). In addition, 24.0% of HCP reported employer requirements for a booster dose. Of those eligible, 90.8% reported receiving a booster vs. 60.2% without a requirement (p< 0.001). Employer requirements for the primary series varied by occupation [46.1% (assistants/aides) to 77.5% (nurses)] and work setting [40.4% (other clinical settings) to 75.4% (hospitals)]. Of 2,157 HCP reporting a primary series requirement, employer accepted reasons for not being vaccinated included medical (56.8%), religious (50.9%), philosophical (10.0%), all three reasons (16.7%), and no reason (14.9%). Factors independently associated with no reported primary series requirements included working as an assistant/aide, in a non-clinical or other clinical occupation, in ambulatory care, long-term care/home health, or other clinical setting, and in the Midwest and South.

**Conclusion:**

Most HCP in this sample reported employer requirements for the COVID-19 vaccination primary series although requirements were less common for boosters. Differences by healthcare setting exist. Implementing workplace strategies, including vaccine requirements, may lead to higher COVID-19 vaccination coverage.

**Disclosures:**

**All Authors**: No reported disclosures

